# Comparative Study of Zirconium Nitride Multilayer Coatings: Crystallinity, In Vitro Oxidation Behaviour and Tribological Properties Deposited via Sputtering and Arc Deposition

**DOI:** 10.3390/jfb15080223

**Published:** 2024-08-13

**Authors:** Julius C. Dohm, Susann Schmidt, Ana Laura Puente Reyna, Berna Richter, Antonio Santana, Thomas M. Grupp

**Affiliations:** 1 Research & Development, Aesculap AG, 78532 Tuttlingen, Germany; 2 Musculoskeletal University Center Munich (MUM), Department of Orthopaedic and Trauma Surgery, Ludwig Maximilians University Munich, Campus Grosshadern, 81377 Munich, Germany; 3 Department of Medical Engineering, IHI Ionbond AG, 4657 Dulliken, Switzerland

**Keywords:** total knee arthroplasty, functional coating, zirconium nitride multilayer, oxidation, pulsed magnetron sputtering, coating tribology

## Abstract

This study aims to evaluate and compare the properties of a biomedical clinically established zirconium nitride (ZrN) multilayer coating prepared using two different techniques: pulsed magnetron sputtering and cathodic arc deposition. The investigation focuses on the crystalline structure, grain size, in-vitro oxidation behaviour and tribological performance of these two coating techniques. Experimental findings demonstrate that the sputter deposition process resulted in a distinct crystalline structure and smaller grain size compared to the arc deposition process. Furthermore, in vitro oxidation caused oxygen to penetrate the surface of the sputtered ZrN top layer to a depth of 700 nm compared to 280 nm in the case of the arc-deposited coating. Finally, tribological testing revealed the improved wear rate of the ZrN multilayer coating applied by sputter deposition.

## 1. Introduction

With a projected increase of total knee arthroplasty (TKA) surgeries of up to 3 million procedures by 2030, the number of revision surgeries due to allergic reactions to implant materials and wear is projected to simultaneously increase, as 2–8% of the general population shows hypersensitive cutaneous reactions to metals such as nickel (Ni), cobalt (Co) or chromium (Cr) [1,2,3,4,5,6]. These implant-related hypersensitive reactions encompass skin changes, delayed wound or bone healing or implant loosening [7,8]. In vitro studies of the uncoated femoral and tibial components disclosed pitting and uneven wear, which originated from the cobalt-rich matrix material and was measured using high-resolution inductively-coupled-plasma mass spectrometry [9].

As a result, coatings have been introduced with the aim to reduce wear and to prevent allergic reactions [10,11]. Clinically proven materials used for tribological coatings include zirconium nitride (ZrN), titanium nitride (TiN), titanium niob nitride (TiNbN) or diamond-like carbon (DLC) [12,13,14]. Deposition of these coating materials is achieved using either chemical vapour deposition (PACVD) or physical deposition (PVD)—more specifically cathodic arc deposition. However, problems with monolayer PVD coatings have been reported in the literature, where an ablation of the monolayer occurs due to the weak elastic modulus [15]. The advanced surface (AS) coating from Aesculap AG is based on an arc multilayer approach. It has been proven successful in maintaining its integrity under high-demanding activity (HDA) knee wear, with no signs of delamination or scratches in articulating areas [3]. Furthermore, a reduction in immunological triggering reactions (IL-8 and IL-10) in ZrN multilayer coated implants was observed when compared to uncoated CoCr implants [16]. Finally, clinical data of the ZrN multilayer coating supporting the successful long term clinical service in patients dates back to 2006 [17,18].

Currently the ZrN multilayer coating method is based on cathodic arc deposition, resulting in surface defects (droplets and pinholes) requiring secondary surface polishing to improve smoothness and reduce surface oxidation. This surface oxidation can cause a modified optical appearance [19]. This has also been observed on retrieved explants coated with the AS multilayer coating, where the optical appearance changed to a greyish discolouration due to surface oxidation, shifting away from the original bright gold-coloured ZrN coating [20]. Thus, an alternative advanced approach to arc coating is based on pulsed magnetron sputtering (MS) has been proposed [21]. Pulsed magnetron sputtering involves sputtering the target material using a plasma rather than vaporizing the target material as in the case of cathodic arc deposition; hence, the multilayer coating is smoother with less pinholes and droplets, ideally leading to a greater oxidation resistance [22].

Due to the need of secondary polishing on concave surfaces with the current ZrN cathodic arc method, applications were not possible without a significantly increased effort. With these advantages in the macroscopic surface of the sputter coating, i.e., the reduced number of defects/pinholes visible, the scope of possible applications can be broadened. New applications can include concave surfaces, such as artificial hip socket inserts or reverse shoulder implant bearings, which can be completely coated with a ZrN multilayer coating. These new fields of applications would be beneficial to patients suffering from metal hypersensitivity caused by CoCrMo in metal-on-metal (MoM) hip replacements. Previous research has linked the increased release of CoCrMo debris found in the surrounding fluids and remote tissues and organs of patients with MoM hip replacements to inflammatory responses and implant loosening [23,24].

The aim of this research was to assess the novelty and advantages of using MS to deposit a ZrN multilayer coating which is currently applied using arc deposition and explore advantages of the deposition process to reduce oxidation of the coated surface. Furthermore, the advantage of reducing the post-coat polishing efforts was explored and basic verification tests were conducted. 

## 2. Materials and Methods

### 2.1. Sample Preparation

**Figure 1:**
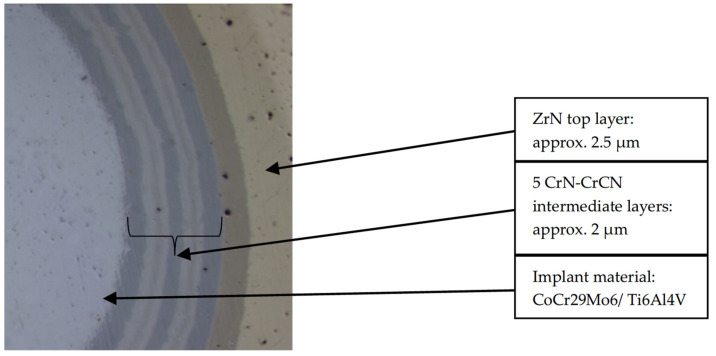
Overview of the structure of the ceramic multilayer coating on the implant substrate.

A total of 18 flat test samples made of either CoCr28Mo6 (material composition according to ISO 5832-12:2019 [25]) (CoCrMo) or Ti6Al4V (material composition according to ISO 5832-3:2021 [26]) (TAV), with a diameter of 20 mm and thickness of 5 mm, were initially surface grinded and subsequently polished to a R_a_ < 0.05 μm. These test samples represented the same material used for commercial implants where the ZrN coating is currently applied using an arc process. Samples were cleaned in a water-based alkaline bath including ultrasound and de-ionized water rinse baths and dried in hot air. Prior to coating deposition, these coupons were pretreated by argon ion etching in cases where sputter deposition was conducted and argon ion and chrome ion etching in cases where arc deposition was conducted. For the ZrN multilayer coating, five alternating intermediate layers of chromium nitride (CrN) and chromium carbonitride (CrCN), and a final zirconium nitride (ZrN) layer were deposited (Figure 1). These intermediate layers help to serve as a bridge between the differing hardnesses and to mitigate residual stress of the softer base material (CCM and TAV) and the hard ZrN top coating. The hardness of the intermediate CrN and CrCN layers were evaluated to 23.3 ± 1.5 GPa and 12.3 ± 1.1 GPa, respectively [27]. In addition, the interfaces between the individual layers act as a diffusion barrier for ions of the base material (CCM or TAV), to mitigatethrough the top layer (ZrN) as well as improving the elastic modulus, as the multilayer coating has a finer crystalline structure compared to a monolayer coating [28]. 

The total thickness of the multilayer coating ranges between 3.5 and 6 µm. Both coating processes featured Ar^+^ and metal ion etching at bias voltages of −600 V and −900 V, respectively. Coating deposition was carried out in an HTC 1200 (Hauzer Techno Coatings, Europe B.V., Venlo, The Netherlands). Technical intricacies of a monolayer chamber setup used for a pulsed sputtering deposition process are detailed in the preceding publication [29]. A similar setup of the coating chamber was used in this research with additional targets of 99.2% Zr (according to ASTM B551 [30]) and 99.5% Cr (according to ASTM 481B [31]) allowing for the consecutive deposition of the Cr- and Zr-based layers forming the ZrN multilayer. Melting and evaporation of the different target materials was carried out via an arc in the case of cathodic arc deposition. The cathodic arc multilayer was deposited utilizing cathode currents of 100 A and 130 A in nitrogen for the Cr- and Zr-based layers, respectively. On the other hand, sputtering of the different target materials was used in the case of the pulsed sputtering deposition process. Pulsed sputtering was conducted at an average target power of 12 kW for the Cr- and Zr-based layers in N_2_/Ar mixtures of 0.5 and 0.2, respectively. 

### 2.2. Oxidation and Characterization

**Figure 2:**
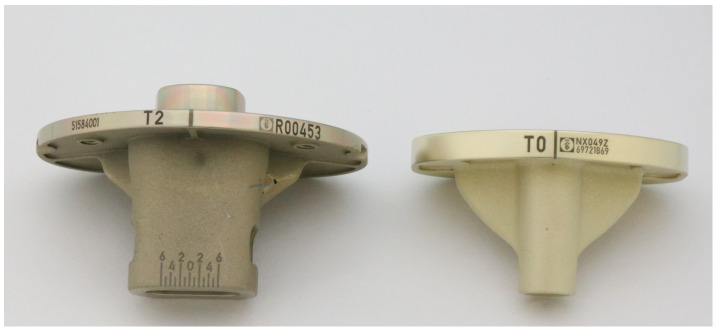
Retrieved oxidized AS arc-coated EnduRo tibial component on the left-hand side and new out of the box ZrN arc-coated tibial component on the right-hand side.

To estimate the surface oxidation of the coating, a retrieved EnduRo (Aesculap AG, Tuttlingen, Germany) tibial explant with an in situ time of 5 years, which had been AS arc-coated (R63 from Schierjott et al. [32]), was analysed by energy-dispersive X-ray spectroscopy (EDX). Here, an oxygen content of 50.2 ± 0.8 at.% was measured for the discoloured surface on the left in Figure 2 [20].

To reproduce in situ coating surface oxidation on the flat coated samples, the samples were subjected to accelerated oxidation. Here, the coated samples were initially cleaned in an ultrasonic bath with ethanol for 30 min and rinsed with distilled water. Accelerated oxidation was carried out by submerging the test samples individually in containers containing 0.9% NaCl (40 mL). The containers were closed with a lid and placed into a furnace (Binder FP400, Binder GmbH, Tuttlingen, Germany) at 90 °C for a total of 10 days. Overview images were taken using a digital microscope (VHX-5000, Keyence Corporation, Osaka, Japan) at the start of the test (initial) and from the oxidized samples after 10 days. To confirm the oxidation process, EDX measurements (X-MaxN 50, Oxford Instruments, plc, Abingdon, UK) were conducted at 12 random spots across the test sample surface at a magnification of 1000× and an operating voltage of 10 kV to determine the oxygen content. Subsequent to the accelerated oxidation, 3 Rockwell indentations per sample were carried out at 1471.5 N and performed according to scale C (Units HRC). The resulting indents were observed using an optical microscope at 100× magnification.

### 2.3. Coating Characterization

The coatings were characterized regarding their surface roughness using a 3D confocal laser microscope (OLS4100, Olympus GmbH, Hamburg, Germany). Measurements were based on the profile method, where the parameters for the calculation were λc = 800 µm and λs = 2.5 µm. 

Scanning electron microscopy (SEM) with EDX was performed using a Carl Zeiss AG, Basic Unit EVO 50 XVP at an acceleration voltage of 10 kV, and 1000× magnification was used for better comparison of the surface features. 

In addition, the crystalline structure of the deposited layers was characterized using X-ray diffraction (XRD) and compared to reference peak datasets using the software Match! (version 3.16). θ to 2θ scans were carried out using a Rigaku SmartLab^®^ equipped with a Cu Kα source operated at 45 kV and 200 mA. The XRD step sizes and collection times per step were 0.02°2θ and 0.12 s, respectively. Utilizing the Scherrer equation, the crystallite sizes within the columnar grains were computed through measurement of the peak broadening at full width at half maximum (FWHM).

The coating composition at the surface and in depth was extracted from X-ray photoelectron spectroscopy measurements (XPS, VersaProbe II, Ulvac-PHI Inc., Kanagawa, Japan) using monochromatic Al(Kα) X-ray radiation (hν = 1486.6 eV). XPS survey scans and core level spectra were acquired from as-coated and oxidized-coating samples and after sputter etching with a 4 keV Ar^+^ beam. The Ar^+^ beam was rastered over an area of 2 × 2 mm^2^ at an incidence angle of 45°. Charge compensation was applied throughout the measurements. The etch depth per time unit was assessed by sputter etching a SiO_2_ wafer and subsequent sdtep height measurements by confocal microscopy. Correspondingly, the data are shown in [nm-SiO_2_]. The composition of the coatings was extracted from core level spectra after subtraction of a Shirley-type background. Here, elemental cross sections provided by CasaXPS software (version 2.3.26) were used. For the deconvolution of the spectra Voigt peaks, shapes with a Lorentzian contribution of 30% were used. The full width at half maximum (FWHM) of all components was restricted to 1.8 eV. 

### 2.4. Tribological Test

For initial tribological testing, reciprocating pin-on-plate tests (Tribotechnic) were used. The afore-specified flat coated CoCrMo test samples were post-coat polished to a surface roughness of Ra < 0.05 μm and cleaned in isopropyl alcohol (IPA). Both coatings were tested against Al_2_O_3_ (RB-6/G25-Al_2_O_3_), ZrO_2_ (RB-6/G10- ZrO_2_) and 100 Cr6 (RB-6/III) balls (i.a.w. DIN5401) with a diameter of 6 mm in ambient, dry conditions using a load of 4 N. The coating wear rate was determined by evaluation of the wear path using confocal microscopy (nanofocus, µSurf EXPERT and μsoft analysis). After scanning across the wear path, its width and depth were determined. Additionally, the wear volume was determined using confocal microscopy. The data from both methods were used to calculate the coating wear rates and corresponding measurement errors. The application of a reciprocating wear test does prevent the application of UHMWPE as counter body material as PE is found to harden under such conditions preventing meaningful results.

## 3. Results and Discussion

### 3.1. Oxidation and Surface Characterisation on CoCrMo Substrate Material

**Figure 3:**
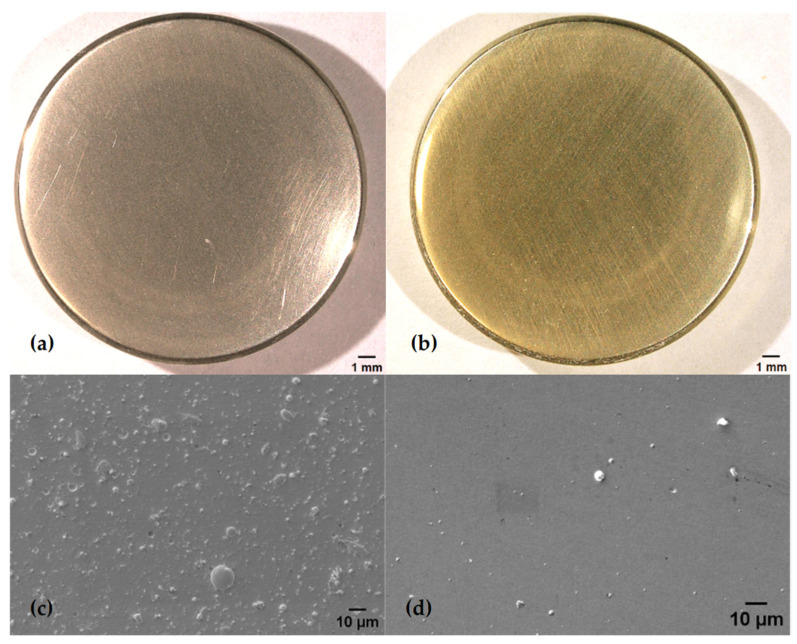
Appearance of coatings prior to accelerated oxidation deposited by (**a**) arc and (**b**) pulsed sputtering methods. Representative SEM plan-view micrographs showing the morphology of (**c**) the arc-deposited coating and (**d**) sputtered coatings at 1000× magnification.

Images presented in Figure 3 show cathodic arc- and sputter-coated test samples after 10 days of accelerated oxidation. Here, a clear shift in the optical appearance compared to the initial colour of the ZrN coating is recorded, indicating the successful artificial oxidation of the ZrN coating. A visible difference in the initial optical appearance of the ZrN multilayer is observed with the sputter-deposited coating displaying a deeper golden colour compared to the arc-coated samples. Similar observations were made by Sproul (1983) in his work on the reactive pulsed sputtering rate for a group IVb nitrides, where a change in stoichiometry for ZrN caused a colour change from bright gold to a darker gold [33].

In comparing the appearance of arc-deposited and sputtered coatings a visual difference in colour is observed, where the arc-coated sample is bright gold and the sputtered sample is golden. Likewise, the plan-view SEM images reveal a clear contrast in roughness. The arc-deposited coating (Figure 3c) shows more emitted droplets compared to the much smoother sputtered coating with fewer droplets and defects present (Figure 3d). The formation of these droplets, as researched by Johnson and Randhawa (1987), originates from local high melting points, the low vapor pressure of zirconium (nitride) and the induced zones of shadow, as the droplets cover Zr on the surface and prevent any further reactions with nitrogen [34]. In contrast, observations made by Ehiasarian and Hovsepian (2004) and Purandare and Ethiasarian (2014) describe a pulsed sputtering mechanism leading to a lower mobility of the Zr ions, resulting in no large-scale defects [21,29]. For coating of medical implants, pulsed sputtering thus produces a surface with the potential benefit of reducing post-coat polishing efforts without a compromise in the surface topography. The difference in topography due to the coating mechanism are corroborated by the measured average surface roughness of the coated coupons as-deposited, R_a_, which for arc-coated samples is 0.08 ± 0.005 μm compared to 0.03 ± 0.005 μm for sputter-coated samples. 

**Figure 4:**
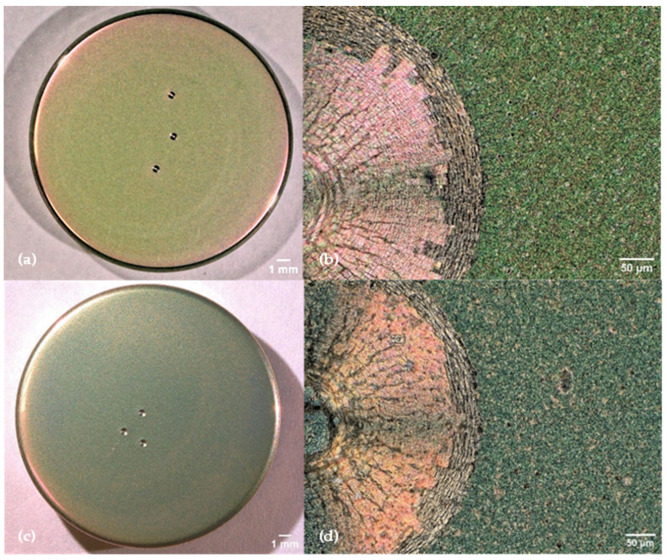
Appearance of coatings deposited by (**a**) arc and (**c**) pulsed sputtering methods after 10 days of accelerated oxidation including HRC Rockwell indentations. Representative optical micrographs of Rockwell indentations at 500× magnification of (**b**) the arc-deposited coating and (**d**) the sputter-deposited coating.

The adhesion of the coatings to the implant material, after accelerated aging of both coatings, was tested using Rockwell C tests carried out with 1471.5 N. These revealed small crack initiation with no delamination of the coating (cf. Figure 4b,d). The lack of delamination can be traced back the absence of surface contamination, facilitating coating adhesion and leading to polycrystalline substrate grains [35]. Table 1 presents the average elemental composition of the ZrN top layer before and after oxidation, with individual measurements taken at the top, centre and bottom of the coupon and a total of 12 individual measurement points across the sample.

A comparison of the chemical composition obtained by EDX between the arc-deposited and sputtered ZrN top layer (Table 1) showed no significant difference, as similar oxygen percentages are measured prior to and after 10 days of artificial oxidation for both coatings. As-deposited, the ZrN coatings show a surface oxygen content of approximately 3 at.%, and after accelerated oxidation the oxygen content is measured to approximately 55 at.%. This shows that accelerated aging of 10 days is sufficient to meet an in situ implantation duration of at least 5 years, which measured 50.2 ± 0.8 at.% [20]. The EDX results provide information from the ZrN top layer, as the ZrN top layer shows a thickness of at least 1.4 µm and the information depth measured by EDX is estimated to be approximately 1 µm. This is corroborated by the fact that no Cr was detected even though the layer beneath ZrN contains Cr. 

The formation of ZrO_2_ is based on the following reaction as described by Harrison (2015) [36]:

(1) $$\mathrm{ZrN}\;+\;O_{2}\;\to \;\mathrm{Zr}O_{2}\;+\;\frac{1}{2}N_{2}$$

The mechanism of oxidation is initiated by oxygen diffusing through grain boundaries at the surface of the ZrN layer; therefore, it is a diffusion-controlled mechanism that occurs as a result of grain boundary fractures [36]. In this study, the grain sizes calculated from the XRD measurements range between 200–250 nm and 120–200 nm for arc- and sputter-coated samples, respectively. Decreasing the clustering in the vapor phase and the bicollision of ions are the two major mechanisms that result in the smoother surface of sputter-coated samples, as has been described by Kateb and Hajihosein (2019) [37].

### 3.2. Oxidation Penetration of Multilayer Coating

**Figure 5:**
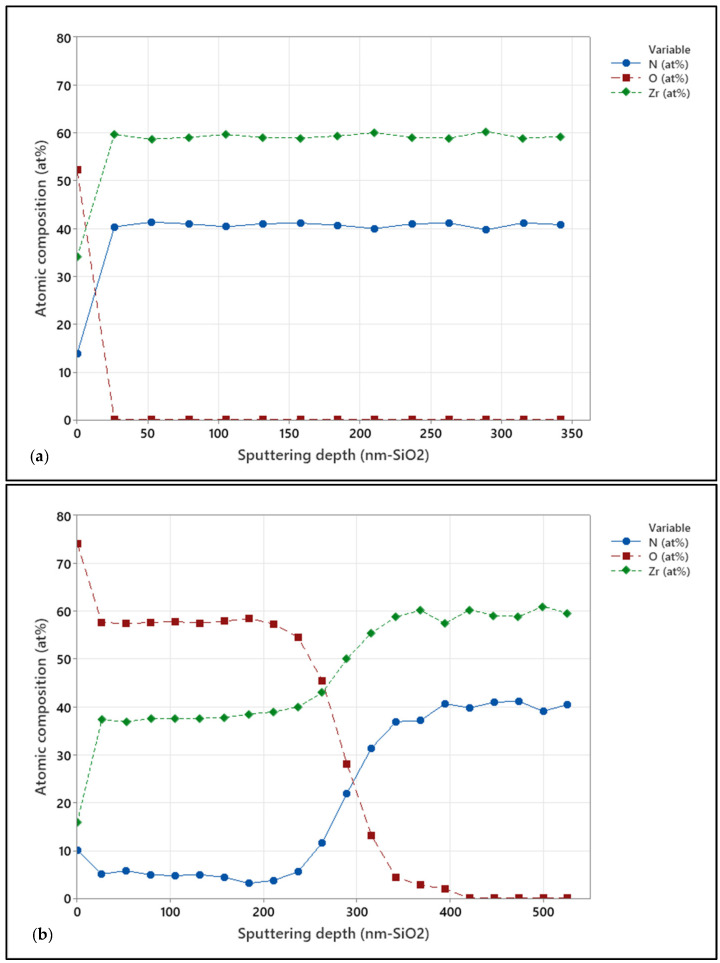
XPS depth profile for the arc-coated CoCrMo samples in (**a**) as-deposited condition and (**b**) after 10 days of in vitro oxidation.

**Figure 6:**
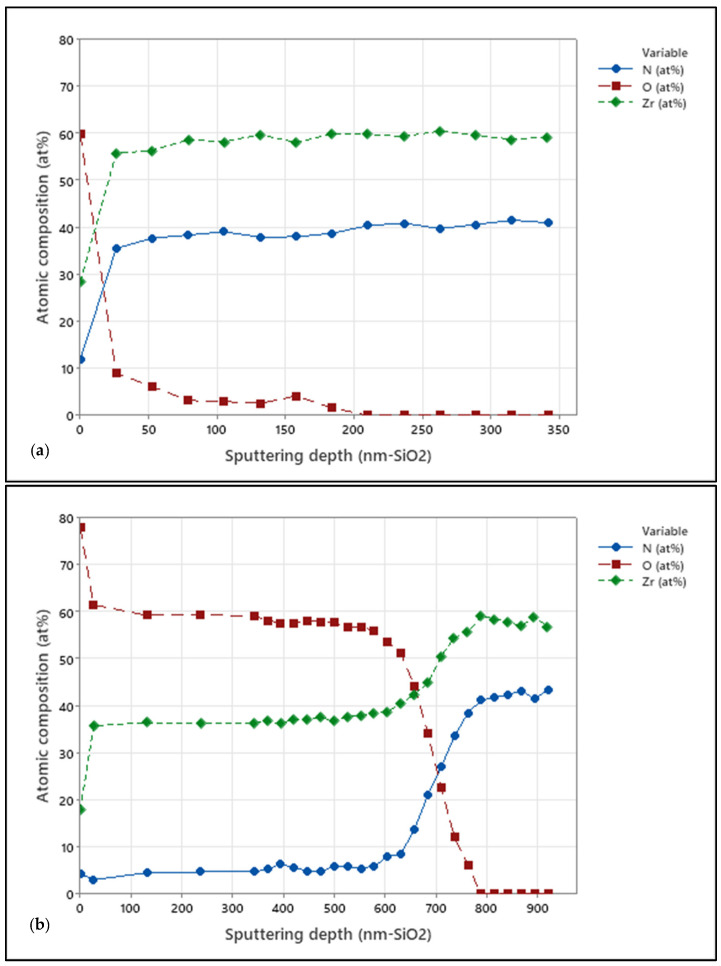
XPS depth profile for the sputter-coated CoCrMo samples in (**a**) as-deposited condition and (**b**) after 10 days of in vitro oxidation.

The effect of grain size on oxide penetration was assessed using XPS depth profiles. Arc-deposited and sputtered coatings on CoCrMo samples in the as-deposited form and after 10 days of accelerated oxidation were investigated. Figure 5 presents the XPS depth profiles for arc-coated samples. Figure 6 shows XPS depth profiles for sputter-coated samples.

Evident is the absence of O in the surface-near region of approximately 50 nm depth of the as-coated arc-deposited samples (Figure 5a), while the sputtered samples show an O content of approximately 3 at.% in the surface-near region and up to 150 nm in depth. After 10 days of accelerated oxidation, the oxygen content of both coatings was elevated to approximately 60 at.% in the surface-near region and up to 280 nm and 700 nm deep for the arc-coated and sputter-coated samples, respectively. This can be ascribed to an increased number of grain boundaries induced by the smaller grain sizes, as in the case of the sputtered coating. This in turn enables increased oxygen diffusion. Similar observations were made by Harrison et al., who observed extensive grain boundary oxidation resulting in a ZrO_x_N_y_ phase and described the oxidation process as being diffusion-controlled [36].

### 3.3. Effect of Coating Process on Crystalline Structure 

**Figure 7:**
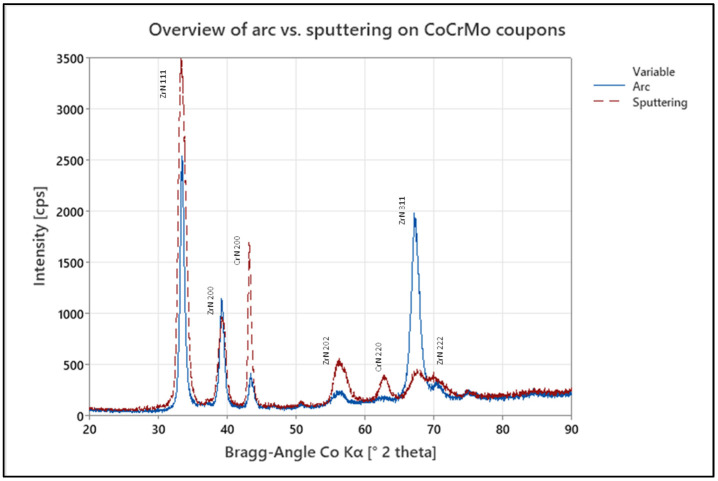
Overview of the XRD pattern of arc- (blue solid line) and sputter-coated (red dashed line) CoCrMo samples as-deposited.

The resulting coating properties are summarized in Table 2 for the two coating processes. To compare the resulting crystalline structure, CoCrMo samples coated with the ZrN multilayer coating using the arc or the pulsed sputtering process, respectively, were analysed by XRD. The resulting XRD patterns of both samples are presented in Figure 7 and show a typical cubic phase structure. The XRD data show the same reflections, but a difference in intensity of the individual peaks. All peaks were attributed to the top two layers of the ZrN multilayer coating with sharp peaks implying a high crystallinity of the ZrN phase.

The coatings deposited by the cathodic arc method reveal a crystalline structure in the (111) and (200) plane, with an FCC spatial arrangement as matched to the crystallography open database [38]. For the sputtered coating, a greater peak intensity in the (111) plane and a lower intensity in the (200) plane can be observed. Similar observations have been made in the literature on TiN thin films, where the preference of orientation was described to be based on strain and surface energies, with lower temperatures resulting in a preferred orientation (100) based on the lowest overall energy conditions [39]. The higher degree of ionization in the arc process is therefore believed to result in a preferred (200) growth. 

### 3.4. Influence of Substrate Material

**Figure 8:**
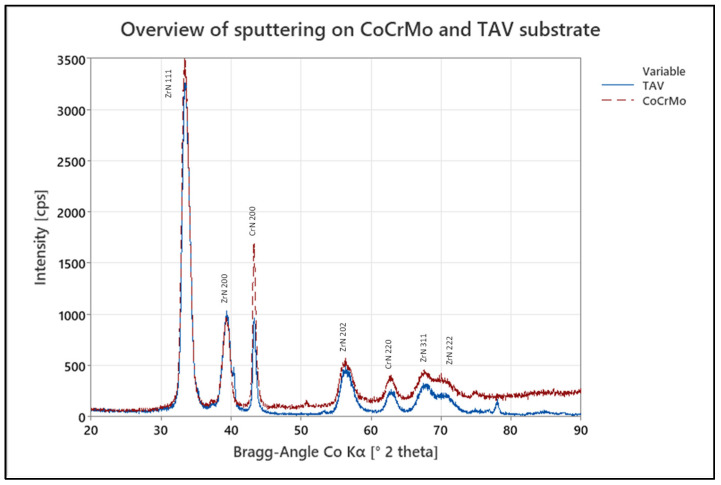
XRD pattern of TAV (blue solid line) and CoCrMo (red dashed line) sputter-coated samples as-deposited.

To assess for the influence of the substrate material on the crystal growth of the multilayers, coupons made of either CoCr28Mo6 or Ti6Al4V were coated with the sputter process. A resulting XRD overview of both samples can be seen in Figure 8, where no significant difference is visible in their respective peak distribution. However, there is an increased level of background scattering for CoCrMo samples. This is due to fluorescence radiation of the substrate material (CoCrMo), which is corroborated by the increase in background intensity at increasing diffraction angle 2θ, as the penetration depth into the base material is increased by the increasing angle.

**Figure 9:**
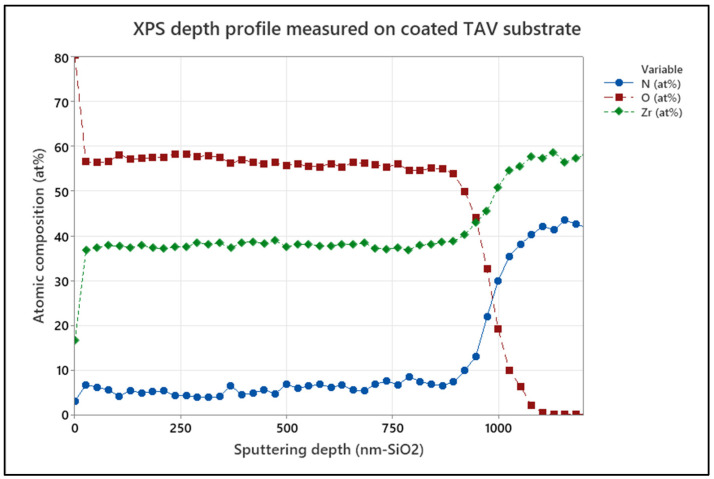
XPS depth profile for sputter-coated TAV samples after 10 days of artificial oxidation.

The comparison of the XPS depth profiles for the oxidized sputter coatings deposited on TiAlV and CoCrMo revealed a slight difference in oxide penetration depth. Comparing the O [at.%] signal in Figure 6b and Figure 9, a difference can be observed for coatings deposited on CoCrMo and TiAlV substrate materials. Here, the oxygen content of both samples was elevated to approximately 57 at.% in the surface-near region with depths approximately up to 800 nm and 600 nm for the sputter coating deposited on TiAlV and CoCrMo, respectively. In contrast, the O-penetration depths upon accelerated aging, as obtained from XPS depth profiles for the arc-coated TiAlV and CoCrMo samples, did not show a significant difference and were calculated to approximately 250 nm. This is supported by the XRD results which showed no significant difference in respective peak distribution and intensities of crystal orientation, indicating that the choice of substrate material does not provide a significant influence on the oxidation penetration of the ZrN multilayer.

### 3.5. Tribological Results

**Figure 10:**
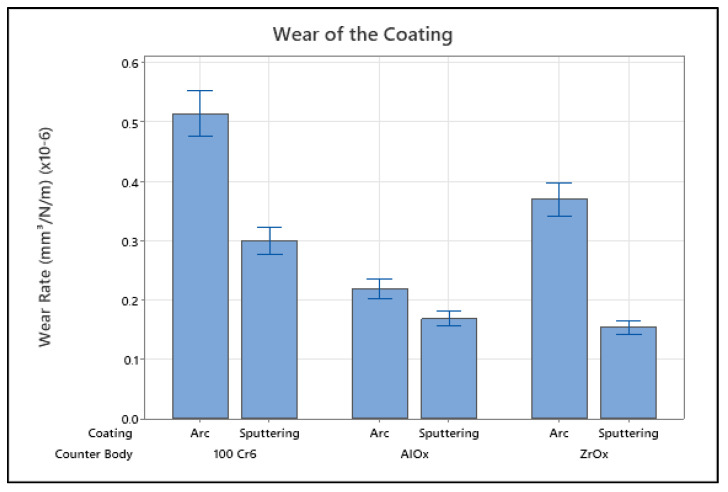
Tribological wear rates of the ZrN multilayer coatings vs. different counterpart materials for both arc- and sputter-coated coupons.

Before carrying out basic application verification tests, ZrN multilayer films deposited on CoCrMo plates either by pulsed sputtering or the arc process were tested to evaluate them for tribological differences. As shown in Figure 10, a measured difference in the wear rate becomes visible between arc- and sputter-coated samples with arc-coated samples having a higher wear rate independent from the counter body material. Furthermore, the lowest wear rate was recorded for the pairing between sputter-coated ZrN and ZrO_x_, whereas the material combination with the highest wear rate recorded was between arc-coated ZrN and 100 Cr6. Although the test set-up is not clinically relevant, these initial wear results favour the sputter-coated samples, as less coating wear was recorded.

### 3.6. Basic Application Verification

**Figure 11:**
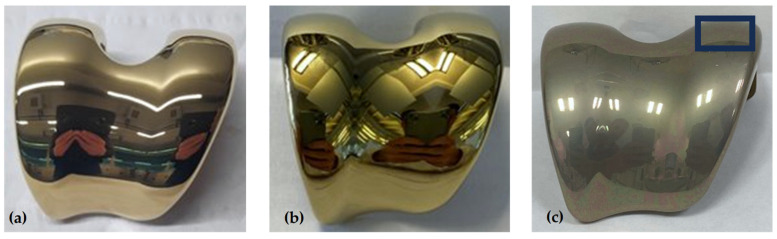
ZrN-coated femoral implants coated by the (**a**) arc and (**b**) pulsed sputtering processes. Image (**c**) shows the oxidative discolouration after 10 days of artificial oxidation of the sputter-coated implant from (**b**) with the marked space on the condyle indicating the area of the ball crater measurements.

For application verification, the ZrN multilayer was applied to CoCrMo femoral implants using the arc process (Figure 11a) or pulsed sputtering process (Figure 11b). Figure 11c shows the sputter-coated femoral component after oxidation, with a clear optical shift away from the deep golden colour towards a greyish colour. This is in line with observations made by Dobosz and Golaszewska (2005), where a colour shift of the ZrN from yellowish-gold to dark blue occurred under high-temperature-induced overgrowth [40]. An oxygen content of 50 at.% is measured across the oxidized sample, indicating successful artificial oxidation, which was carried out using the same artificial oxidation process as previously described for the coupons.

**Figure 12:**
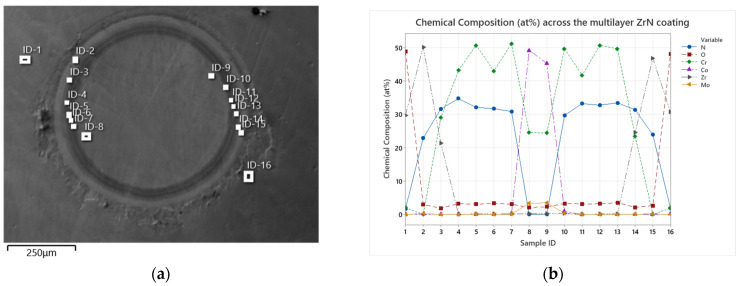
(**a**) Overview of the ball crater created on the articulating condyle of the oxidized femoral implant with the individual sublayers of the multilayer visible as well as marked with individual measurement points. (**b**) The chemical composition as atomic weight (%) measured at the individual points referring to the thin adhesive Cr bond layer, the five alternating intermediate layers of alternating CrN and CrCN layers, the transition layer and finally the ZrN layer, which marks the outer most layer with the oxidative discolouration.

The chemical composition across the ZrN multilayer coating measured on a crater made at the condyle surface (black box shown in Figure 11c) can be seen in Figure 12.

A clear separation of the multilayers can be seen both under the microscope (Figure 12a) as well as from a chemical composition point of view (Figure 12b), with an increased oxygen at.% only measured in the final ZrN layer at both ID-1 and ID-16. In addition, a smooth surface can be observed under the SEM, with little to no defects present around the ball crater, confirming the improved surface texture of the ZrN coating applied with the sputter process from the preceding SEM images in Section 3.1. When referencing back to the retrieved EnduRo (Aesculap AG, Tuttlingen, Germany) tibial explant with an in-situ time of 5 years, which had been arc-coated (R63 from Schierjott et al. [32]), a similar surface discoloration is observed with the sputter-coated femoral implant. Moreover, a measured surface oxygen content of 49.8 ± 0.7 at.% is measured on the sputter-coated femoral component compared to 50.2 ± 0.8 at.% measured for the discoloured arc-coated EnduRo tibial explant, implying a very similar level of surface oxidation. Thus, contrary to initial expectations, no reduction in the surface oxidation and the associated discolouration of the ZrN layer could be achieved with the sputter process.

## 4. Conclusions

This study evaluated and compared the properties of a zirconium nitride (ZrN) multilayer coating prepared using two different techniques: pulsed magnetron sputtering and cathodic arc deposition. The findings reveal that the pulsed sputtering process results in a distinct crystalline structure with smaller grain sizes compared to the arc process. These smaller grain sizes increase the number of grain boundaries, enhancing the surface area for oxygen diffusion, which may lead to a deeper oxygen penetration within the zirconium layer. Despite differences in oxygen uptake, both deposition techniques demonstrate similar coating adhesion, and the substrate material exhibits minimal influence on the crystalline structure, grain size and oxygen uptake, highlighting the predominant role of the coating process itself. Moreover, tribological results indicate that the ZrN coating applied via pulsed sputtering exhibits an improved wear rate, confirming its suitability for applications on concave surfaces such as femoral knee implants.

## 5. Patents

A Patent has been filed related to this topic with following title “Wear-reducing hard material multilayer coating on concave geometries of medical implants”.

## Figures and Tables

**Table 1:** Average elemental composition in atomic percent, measured by EDX, of the as-deposited coatings and the coatings after 10 days of artificial oxidation.

	N [at.%]	O [at.%]	Zr [at.%]	Total [at.%]
Arc (0 days)	44.8 ± 0.6	3.8 ± 0.8	51.4 ± 0.8	100.00
Pulsed Sputtering (0 days)	46.2 ± 0.5	2.7 ± 0.3	51.1 ± 0.4	100.00
Arc (10 days)	10.7 ± 0.5	53.0 ± 1.1	36.3 ± 0.9	100.00
Pulsed Sputtering (10 days)	8.7 ± 0.9	59.5 ± 1.1	31.8 ± 1.0	100.00

**Table 2:** Summarized coating properties of arc- and sputter-deposited ZrN multilayer coatings on CoCrMo samples.

	Average Surface Roughness, R_a_ [µm]	Rockwell C Adhesion [ISO Class]	N/Zr Ratio by EDX	ZrN Coating Thickness [µm]	Grain Size by XRD [nm]	Coating Wear Rate against ZrO_x_ [mm^3^/N/m]	Appearance
Arc coating as deposited	0.08 ± 0.005	1	0.87	1.6	200–250	3.7 × 10^−6^ ± 0.5 × 10^−6^	Bright gold (14k)
Sputtered coating as deposited	0.03 ± 0.005	1	0.90	1.4	120–200	1.5 × 10^−6^ ± 0.2 × 10^−6^	Gold (22k)

## Data Availability

The authors do not have permission to share data.
